# Altruism in Paramedicine: A Scoping Review

**DOI:** 10.3390/healthcare10091731

**Published:** 2022-09-09

**Authors:** Leigh Parker, Sarah J. Prior, Pieter J. Van Dam, Dale G. Edwards

**Affiliations:** 1 Tasmanian School of Medicine, University of Tasmania, Hobart, TAS 7000, Australia; 2 Tasmanian School of Medicine, University of Tasmania, Cradle Coast Campus, Burnie, TAS 7320, Australia; 3 School of Nursing, Cradle Coast Campus, University of Tasmania, Burnie, TAS 7320, Australia

**Keywords:** altruism, paramedicine, caring, compassion, caring science, paramedic education

## Abstract

While altruism has been studied in healthcare professions such as nursing and medicine, the exploration of the characteristics of altruism, as related to paramedicine and emergency care in Australia, is limited. This scoping review explores altruism in paramedicine from the perspective of the paramedic as practitioner, learner, and educator as seen through the lens of the paramedic and the patient. Also discussed is the positive impact of altruism on the patient experience of care. A scoping review was used to assess the availability of data related to altruism in paramedicine. The Preferred Reporting Items for Systematic Reviews and Meta-Analyses extension for Scoping Reviews was used to guide the process. Search categories were orientated around the subject (altruism) and discipline (paramedicine). A total of 27 articles are included in this scoping review. Initial searching identified 742 articles; after duplicate removal, 396 articles were screened with 346 excluded. Fifty articles were full-text reviewed and 23 excluded. The final 27 were extracted following full-text screening. None of the articles are specific to altruism in paramedicine. The data related to the practice of altruism in paramedicine are extremely limited. The preponderance of data arise from Europe and North America which, due to crewing and service differences, may impact the practice of altruism in different regions. Recent changes to the scope of paramedic practice, workload, education, and case acuity may influence behaviour regarding altruism, compassion, caring, and associated caring behaviours. The practice and education of paramedics including altruism, compassion, caring and caring behaviours in the Australasian setting warrants further research.

## 1. Introduction

Altruism is the behaviour of caring for others without seeking self-gain and, in healthcare, is the act of putting patient interest ahead of self-interest. Altruism is described by Batson [1] as an important force in human affairs, a motivational state based on nurturance. Altruism is a component of professionalism alongside accountability, excellence, duty, honour and integrity and respect toward others [2]. In nursing literature, altruism has been described as ‘the heart of nursing’ and is associated with deep respect, dedication to service and promotion of another’s welfare, and compassion that puts patient interest to the fore [3]. Ideally, an altruistic act in a caring situation stems from an authentic wish to alleviate suffering [4]. The act of benefitting the self through altruistic acts for others is not altruistic; motivations that stem from self-interest may affect the consistent and predictable delivery of care [3]. This is not to say that there is no benefit to the carer derived from the practice of altruism. It is widely recognised that “reasonable altruism”, defined by Post as helping behaviour that is not overwhelming, can result in improved wellbeing, health, happiness, and longevity [5]. Further, compassion, a positive humanising trait, is associated with altruism [6] and defined by an awareness of another’s suffering and a desire to resolve it. Compassion plays a role in healthcare through the motivation to relieve other’s suffering through taking action [7]. Caring interactions prioritise the patient perspective, viewing the person as able to make, and be responsible for, choices; these connections are humanistic and altruistic. Authenticity, patient focus, emotional presence and the promotion of patient well-being are behaviours associated with caring [8]. Similarly, empathy is one’s ability to emotionally understand what other people are feeling, to be able to see things from their viewpoint, and imagine oneself in their shoes [9]. Empathetic distress is the term used to describe a situation where a healthcare worker unintentionally picks up on the distressing emotions of their patients, or families, and may wish to avoid or withdraw from the person who is suffering [10]. It is important to recognise this concept to distinguish it from compassion, a central component of healthcare delivery.

The nature of ambulance work has evolved over time, and continues to evolve, from the provision of emergency transport to incorporating a broader scope of practices that include treatment and discharge in the field and referral to health services while still including more traditional models of out-of-hospital care [11]. These changes to service provision have coincided with an increase in ambulance call volumes throughout the developed world [11]. Alongside increased workloads, models of paramedic education have evolved. Paramedic education in Australia has seen a shift to university-based degree education models [12,13], a broadening of the paramedic scope of practice, and greater responsibility with regard to clinical decision making, as well as treat-but-not-transport situations [12]. This evolution of the paramedic role, and the urgency or non-urgency of a case, may influence the way altruistic behaviours are incorporated into modern paramedicine. Finding an appropriate balance between caring and medical interventions is vital in paramedicine [14] to achieve desirable patient outcomes.

The aim of this scoping review is to explore the role of altruism in paramedicine from an international perspective to gain an understanding of the existing literature and subsequent knowledge gaps. For the purposes of this article and to achieve an international perspective, the term ‘paramedicine’ is used to encapsulate those healthcare professionals delivering out-of-hospital healthcare while operating from an ambulance service. The professional background of those who provide ambulance care differs around the world. In Australasia, the predominant provider of ambulance care is the paramedic [12], whereas across Europe, the composition of ambulance crews varies; for example, an ambulance could be crewed with a nurse, emergency medical technician (EMT), paramedic, or physician [15]. In North America (USA and Canada), emergency medical services (EMS) are staffed by EMTs and paramedics. The term paramedic will be used in this article. It is unclear if differences in practice influence altruism in paramedicine.

## 2. Materials and Methods

A scoping review was utilised to assess the breadth, depth, and type of available research related to the practice of altruism in paramedicine. The overarching guideline for this scoping review is the Preferred Reporting Items for Systematic Reviews and Meta-Analyses extension for Scoping Reviews (PRISMA-ScR) checklist [16]. The framework has five stages: identification of the research question; identification of relevant studies; selection of studies; charting of data; organisation and reporting of results. Subsequent enhancements of this framework by Levac et al. and Peters et al. are presented in the JBI manual for evidence synthesis (JBIMES) Section 11.1.3 [16].

### 2.1. Criteria for Search

#### 2.1.1. Inclusion Criteria

The articles included in this scoping review were those related to altruism in the field of paramedicine. Articles were accepted if published between 2010–2021, in English, and where full text was available. Our search focused on this period to capture contemporary discourse, recognizing the rapid change affecting the field of paramedicine in recent decades.

#### 2.1.2. Exclusion Criteria

Articles that explored caring from the perspective of patient management (i.e., clinical reasoning or intervention) and did not address elements related to altruism were excluded. Articles focused on care within defined physical settings (such as emergency departments) were excluded. Grey literature was excluded. Books were excluded to ensure contemporary commentary on the evidence, recognizing that books may refer to publications outside the inclusion criteria.

### 2.2. Search Strategy

Searches were conducted between August 2020 and December 2020. Initial search terms were divided into two categories—altruism and the discipline of paramedicine.

Category One: Altruistic* OR caring OR mindfulness OR “above and beyond”.

Category Two: ems OR emt OR ambulance AND officer OR paramedic* OR emergency AND medical AND service OR emergency AND medical AND technician.

After searching each category these were then combined to create a third category.

Category Three: Altruistic* OR caring OR mindfulness OR “above and beyond” AND ems OR emt OR ambulance AND officer OR paramedic* OR emergency AND medical AND service OR emergency AND medical AND technician.

The databases included were Scopus, Medline via OVID, CINAHL, PsycINFO and PUBMED.

### 2.3. Selection

Articles were uploaded to Covidence™ systematic review software (Veritas Health Innovation, Melbourne, Australia. Available at www.covidence.org, accessed on 6 April 2022) [17] for screening. The initial screening included a review of the title and abstract. Articles that related to the topic of altruism in paramedicine were retained, including those articles that had content related to the patient experience or the way the paramedic delivered care. Articles that featured a key word (such as care or mindfulness) were assessed for their relevance and eliminated if the topic did not relate to altruism, or caring behaviours, in paramedicine. Nursing-related articles were included if they originated from regions where ambulances are crewed by registered nurses. Within Covidence, two authors independently screened titles and abstracts with a third resolving any conflicts; the same screening method was used for full-text screening.

**Figure 1:**
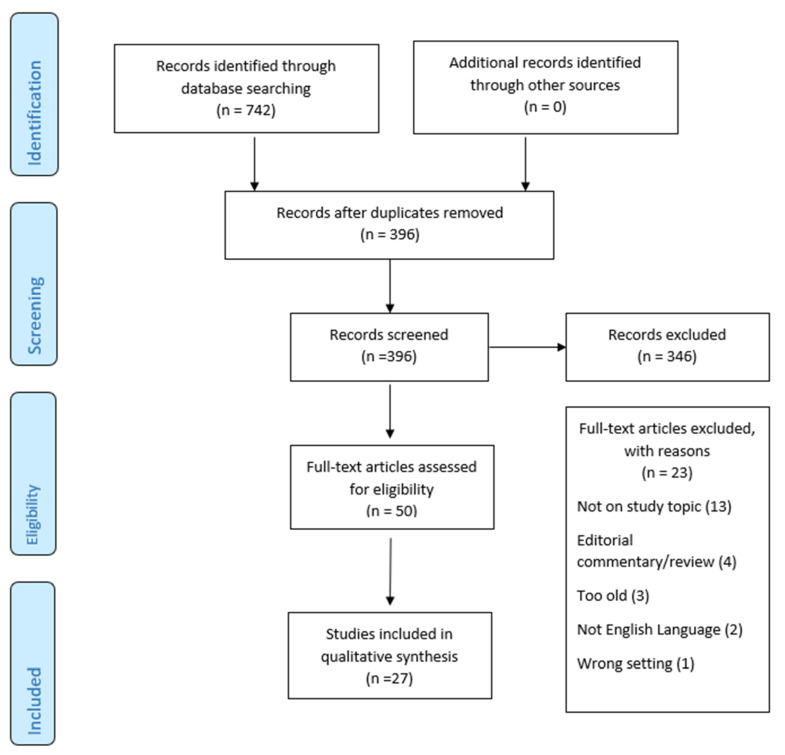
Article Selection Flow Chart.

A total of 27 articles are presented in this scoping review. Of the 23 studies excluded, 13 were not on the study topic; four were either editorial commentaries or reviews; three exceeded the date parameters; two were not in the English language; and the remaining one was deemed the wrong setting. Article selection is presented in Figure 1. Articles for inclusion are presented in Appendix A, Table A1.

## 3. Results

Of the 27 articles included, ten originate from Sweden [14,18,19,20,21,22,23,24,25] and an eleventh from both Sweden and Spain [26]. Five articles originate from the USA [27,28,29,30,31] and another five from the United Kingdom [32,33,34,35,36]. Three articles originate from Canada [6,37,38] while one each hail from Australia [39], Denmark [40], and Norway [41]. Most articles were qualitative studies; however, the findings included a meta synthesis of 12 articles related to self-harm (and the perceptions of those providing care) [35], a systematic review of 16 articles related to self-harm (and the perceptions of those providing care) [36], and a systematic review exploring caring science in the out-of-hospital setting [24].

Five articles were original commentaries, four based on the perspective of caring as seen through an experience viewpoint, and the fifth, respectively, based on both professional and personal experience [6,27,28,30,31]. The paramedic perspective dominated findings; however, five articles presented research regarding the patient perspective of paramedic care [18,21,22,37,40]. Following analysis of the literature, three major themes emerged: caring interactions in the out-of-hospital setting; care impact; and role of paramedic education and/or educators.

### 3.1. Themes

#### 3.1.1. Theme 1: Caring Interactions in the Out-of-Hospital Setting

Twenty-two articles explore aspects of caring interactions in the out-of-hospital setting [6,14,15,18,19,20,22,23,24,25,26,27,28,30,35,36,38,39,40,41] including commentary regarding the complexity and multi-faceted phases of out-of-hospital delivery [14,19,40] and how these phases affect, or are affected by, patient–paramedic interaction [14,18,19,20]. Ten articles explore the nuances of providing care in specific case settings, for example self-harm [33,34,35,42,43], palliative care [38], cancer-patient resuscitation [41], and trauma patients using helicopter medical services [22]; studies were not limited to the patient–paramedic relationship with one also profiling the family/bystander–paramedic relationship [15].

Bremer et al. explored values held by paramedics in Sweden and Spain, finding that both groups favoured utilitarianism least, exploring how this might contrast with the values of the organisations providing out-of-hospital care [26].

Several articles include commentary on the importance of paramedic education related to the provision of caring [14,19,20,34,36,38].

#### 3.1.2. Theme 2: Care Impact

Twelve articles explored the impact of care on the patient [6,18,22,23,24,27,35,36,37,38,39] and/or bystander(s) [15,38].

#### 3.1.3. Theme 3: Paramedic Education

Seven articles explored paramedic education related to aspects of caring in areas such as: mindfulness [29], palliative care [38], emotional labour [32], self-harm [33,35], core values [31], empathy [39], and caring science [24].

## 4. Analysis and Discussion

This scoping review aimed to explore the characteristics of altruism within paramedicine. Given the paucity of discipline-specific data directly related to the term altruism, behaviours associated with altruism, inclusive of characteristics of compassion and caring, were reported on. Three overarching themes underpinned the findings: caring interactions in the out-of-hospital setting; care impact; and role of paramedic education and/or educators.

### 4.1. Theme 1: Caring Interactions in the Out-of-Hospital Setting

This theme explored the complexities of establishing and maintaining a caring interaction in the out-of-hospital setting. It was not surprising to find that most of the altruistic behaviours were displayed in the out-of-hospital setting, as this is the environment in which paramedics practise. However, to care in the out-of-hospital setting, paramedics need to be prepared and flexible; they need to be certain and in control while being open, understanding that facets of a case may change while on scene [23]. Care needs to be given in the context of the patient’s world and with the understanding that the experience will have future meaning for that patient. Homberg et al. suggest that paramedics need to be “*pliable to patient wishes*” [20]. Thus, caring interactions prioritise the patient perspective and view the person as able to make and be responsible for choices; these connections are humanistic and altruistic. Authenticity, patient focus, emotional presence, and the promotion of patient well-being are behaviours associated with caring [8].

Paramedics need to simultaneously form a caring relationship and provide clinical care [20], noting that (almost) every patient interaction is new, orientated around practices and resources (i.e., equipment), and often unpredictable [40]. Elmqvist et al. explored the interplay between first responders’ expectations of being ‘in the role of the hero’ and ‘being genuine’ in interpersonal interactions and their expectations balanced between an outwardly projected calm overlaying a constant shift between ‘being and doing’ [19] which links back to mindfulness as a key component of caring. The paramedic needs to be personal while in their professional role: The professional role requires the paramedic to be an authority while protecting, respecting, and acknowledging the patient; this is intertwined with the personal characteristics of being emotionally affected, and caring beyond clinical requirements [20]^.^

Togher et al. identified that professionalism and communication contribute to confidence, leading to a sense of reassurance [44]. They describe interpersonal skills such as calmness, kindness, and the ability to inform as positive components of professionalism. Professional calmness was presented in a non-verbal manner that indicated control of the situation [8]. Establishing mutual confidence, and trust, facilitates the delivery of patient-centred care. Effecting a safe situation for open and safe interactions, considering and valuing patient emotions, inviting patients to participate in care planning, active listening, calmness, and collaboration were valued by patients [21]. It was found that care interactions involve focusing on the patient rather than the first responders’ own needs, an unselfish focus on the injured person—a focus enabled by the first responder role, hero costume, and a systematic approach. The role of empathic distress in this situation can be overwhelming as the patient focus often means that the first responders are unaware of their own personal distress. Elmqvist posed the question “are first responders doing in order to be able to be, or are they being in order to be able to do?” [19]. Regarding ‘being,’ it is worth considering how the paramedic positions themself when ‘being’ the paramedic and ‘doing’ paramedicine. Dick suggests that kneeling is a posture used during most cases, a position of convenience and necessity that can facilitate eye contact, instil confidence, and express humility, thereby eliminating barriers between paramedic and patient [28]. Rubin suggests that paramedics should augment their clinical care with conversation, comfort (e.g., pillows and blankets), choice (e.g., music enroute, destination), non-clinical physical contact (e.g., holding a hand), and more assertive pain relief [45].

Three authors noted that ambulance cases have several components [14,19,40], and the way these components progress influences the paramedic–patient interaction [14,18,19] and therefore subsequent experience of care. It is also important to acknowledge the vital first contact.

The care experience starts with the arrival of the ambulance, at which point it is possible to begin to establish trust and confidence—paramedic knowledge and calmness is important, and patients can lose trust and confidence if they feel they have called an ambulance inappropriately [42]. Rees et al. suggest that first contact with patients who self-harm is key to their acceptance or rejection of care and may influence later self-harm behaviours [36], suggesting that the patient impact lasts beyond the paramedic/patient interaction. Effective teamwork can also provide the patient with a positive, soothing experience as identified by Sandstrom et al., who explored patient experiences of helicopter transportation. A sense of being cared for and safe enabled patients to ‘hand themselves over’ to staff and a sense of trust arose from being taken seriously [22], suggesting that all members of a team are integral to a positive patient experience.

Other factors to consider include the need to manage both the patient’s presenting condition and understand the patient’s lifeworld while managing time. Perceived urgency may result in the paramedic focusing on the ‘doing’ at the expense of finding time for calmness. Calmness allows an incorporation of ‘being’ into the relationship—combining the complementary clinical and care sciences to add depth to objective information and facilitate safer patient care decisions through flexibility and correct interventions, as well as limiting patient suffering and worry [25].

Ultimately, out-of-hospital care is the first link in the chain to total care [18] with findings indicating that paramedics need to be aware of patient expectations and world; be able to create calm, trusting environments; and work effectively in a team setting. Paramedics need to be an authority, protective, respectful, and pliable while maintaining the communication required in a patient–paramedic interaction.

### 4.2. Theme 2: Care Impact

This theme explored the impact of the paramedic interaction with the patient on the patient’s experience of care. Excepting work by Elmqvist et al. [19], in-depth patient interviews in the out-of-hospital context are limited [18], which may limit knowledge regarding the patient perspective and impact of care. Ahl and Nystrom explored the potential positive and negative aspects of the paramedic–patient relationship, describing moments from first arrival to patient handover where the relationship can establish or lose trust and confidence, understanding that patient expectations and paramedic calmness and knowledge play roles in acceptance or rejection of care [18].

In a meta-analysis of the literature, Rees et al. found that self-harm patients had negative experiences of healthcare services inclusive of hostile staff responses and limited knowledge; patients felt ignored and perceived as difficult or as time wasters [35]. Rees et al. suggest on-scene factors can impact care; for example, shame and embarrassment felt by the patient may limit information gathering. They added that prior knowledge of the patient could be seen as a positive or negative dependent on whether the paramedic experienced reduced sympathy or case insights through deep knowledge of the patient [36]. Batson et al. [43] implies that valuing the patient would allow the paramedic to adopt the patient’s perspective, being able to imagine how the patient feels and thinks in that moment; as a key component of empathy, Sundstrom and Dahlberg [23] suggest openness and a willingness to listen to, see, and understand the patient is more important in paramedicine than in other areas. This listening is a component of a lifeworld-led care approach which can aid medical assessment and reduce patient suffering. The long-term impact caring interactions may have on the patient has been previously discussed; however, this impact, if negative, may be significant enough to be life-threatening. With self-harm there is a one-year association of self-harm behaviours with the risk of completed suicide [34]—a concern, and particularly so where previous negative experiences reduce the patients willingness to seek further help.

Altruism, compassion, caring, and associated behaviours (such as trust) have been shown to: prioritise the patient’s own perspective [8,18]; facilitate patient-centred care [21] and patient safety [25]; reassure and indicate situational control [44]; instil confidence [28]; improve acceptance of care [18,34]; facilitate a willingness to hand oneself over to care and store positive memories post-incident [22]. The concept of caring science in relation to nursing care has been explored since the 1950s [18]; however, there is a paucity of data relating to caring science in paramedicine, particularly in regions where ambulances are not crewed by nurses.

### 4.3. Theme 3: Paramedic Education

Given that altruistic, compassionate, and caring practice positively impacts patient experience, it is important to consider caring education in paramedicine. This review has identified that paramedic education in mindfulness [29], ethics [34,41], and empathy [39] are important, as is an understanding of student awareness of emotional labour [32]. Ducar et al. found that the introduction of a ‘mindfulness for healthcare providers’ program for EMS personnel significantly reduced burnout and increased compassion, satisfaction, and mindfulness scores [29]. Compassion fatigue, termed empathic distress fatigue, occurs when an individual is emotionally drained due to accidentally sharing the distressing feelings of the patient through emotional contagion, and can affect patients through irritability and reduced standards of care. Mindfulness training has been linked to increasing compassion, patient satisfaction, and care outcomes [29] and could be a useful tool for reducing empathic distress.

Rees et al. identified that the self-harm care interaction was ‘uniquely complex;’ paramedic education is limited as is confidence and competence, yet self-harm is strongly correlated to suicide, giving rise to a situation of ‘wicked complexity’—they describe the need for education in the field of self-harm as urgent [33]. Education in mental health inclusive of values such as caring, empathy, professionalism, non-judgement, non-discrimination, and patient centeredness is considered a priority [35].

When paramedics face conflicting top-down and bottom-up pressures (double pressure situations), the situation can be compounded by the paramedics’ need to make rapid decisions. Top-down pressures include organisational values, whereas bottom-up pressures could arise from the professional standards and observations of the paramedic. Ethical problem-solving guidelines, uniform practices, and education can help with weighing options [46].

Paramedics caring for those who self-harm can experience decision-making difficulties, such as the conflict between the patient’s right to refuse transport and the paramedic’s desire to act in the patients’ best interest. Although the paramedic may be acting in an altruistic manner, the patient may not be ready to receive this altruistic act, particularly in this circumstance. Practical, real-world ethics training can prepare paramedics to make sound ethical decisions [34]; other decision-making challenge areas would likely also benefit. Williams et al. discuss the value of empathy in paramedicine and its benefit to the patient in terms of communication, trust, and positive care outcomes, and suggest that empathy needs to be incorporated into evidence-based teaching curricula [39].

Jennings explored student paramedic awareness of the emotional demands of paramedicine. The author described emotional labour as the work exerted when in the role, and the effort required to maintain the professional affect associated with the role. Compassion was described as the intelligently kind way to deliver care, inclusive of empathy, respect, and dignity. Jennings concluded that a proper understanding of student awareness of emotional labour would facilitate the appropriate incorporation of compassionate care into course curricula [32].

Eaton explored the interplay of evidenced-based practice and values-based practice in paramedic education. Values-based practice was found to develop the understanding of the patient perspective through understanding their values and using this understanding when working toward evidence-based and patient-focused outcomes. During placement time, students become part of the practice community, which may influence their professional values, identity, and behaviours, including student modelling of displayed preceptor behaviour [47]. Given the impact of role-modelling on student development, it is important to ensure that altruism, compassion, and caring is understood and appropriately modelled by educators, including preceptors. Modelling of such behaviours may need to extend beyond teaching requirements, with behaviours modelled in general engagement, for example interacting with the student in an altruistic, compassionate, and caring manner. Young suggests that with experience a paramedic learns when to enact skills such as compassion, listening, and physical contact, and when it is necessary to direct, speak, maintain distance, and move promptly [6]. It would be interesting to explore simulation as a way of helping students learn skills, and when to enact them, while safely growing a student’s level of experience.

Care interactions can extend beyond the patient to include family members. Bremer et al. identified that paramedics understood the need to care for family (post fatal cardiac arrests) but felt inadequate doing so. Care interactions in this situation require shifting from known to unknown frameworks, the ability to respond situationally, and advanced knowledge and ethical caring competence [15]. As such, education needs to extend the exploration of caring for the patient and include caring for family and bystanders. Brainard suggests that it is compassion, rather than medical procedures, that matter most to surviving relatives and friends [28]. Education can positively influence the caring interaction as identified by Carter et al., who reported on family and paramedic perspectives following the implementation of a palliative care program. Paramedics voiced support of the program, reporting increased comfort and confidence when delivering palliative care. Families commented on the professionalism, compassion, and “going above and beyond” with care including family/friends as well as the patient [38]. Brydges et al. [37] explored the perceptions of older adults experiencing community-based healthcare provided by paramedics. They found that paramedics were seen to be caring, respectful, and trustworthy, and that they fulfilled roles as both health advocates and emergency care providers. An incidental finding was that the paramedics working in the community setting were also valued for their emergency skillsets (which were utilised on occasion); the authors suggested this as an added bonus to having paramedics in this type of role in the community role [37]. While over time the provision of ambulance care has diversified, paramedics need a foundational base of competence in life-preserving skills and the ability to work in a wide variety of settings.

Given that clinical confidence may affect a paramedic’s ability to ‘be’ and ‘do’ paramedicine, education in a wide variety of case types and patient interactions is an important factor underpinning caring interactions in the out-of-hospital setting, as is clinical and caring competency.

### 4.4. Limitations

This scoping review aimed to explore the literature related to altruism in paramedicine; however, due to the dearth of literature in the Australasian context, the review incorporated an international perspective. This resulted in the inclusion of research involving a range of health professions other than paramedics, due to the diversity in professional groups delivering ambulance-based out-of-hospital care internationally. This professional diversity may limit the transferability of some data and findings to the Australasian setting.

The intent of this scoping review was to focus on contemporary literature, and as such publications prior to 2010, or those that might draw conclusions from prior to 2010 such as books and reviews, were not included. This may serve as a limitation to the breadth of historical knowledge; however, when considered in the context of rapid professional change, it was deemed appropriate.

This scoping review focuses on the interaction between paramedic and patient. There are, however, other dimensions in which altruism plays a part, including the interaction of the paramedic as an employee with their employer, the paramedic interacting with their wider community, and paramedic-to-paramedic peer interactions. None of the articles identified in this study reflected these interactions. At first glance this may indicate a gap in the literature; however, the search terms utilized in this scoping review may not have been sufficient to capture these interactions.

Research into the practice of altruism in paramedicine (and associated behaviours) in the Australasian setting is warranted. This includes research into both the paramedic and patient perspective of altruism.

## 5. Conclusions

Key to altruistic practice is the willingness to prioritise the patient over oneself without seeking self-gain. Compassion and caring involves the ability to ease negative patient experiences such as suffering and anxiety, and to reduce vulnerability through behaviours such as calmness, along with a focus on the patient as a person with a unique story, values, and needs, creating a sense of mutual trust. Data related to the practice of altruism, compassion, and caring in paramedicine are extremely limited; where data are found they tend to originate from the northern hemisphere, particularly Sweden. Ambulance crewing arrangements differ internationally, which may limit transferability of data to the Australasian setting. In recent years there have been significant changes to the way paramedics practice, including more advanced skillsets, greater case range (low to high acuity), and increased clinical decision-making responsibility. Ambulance services have seen increased demand over a wide range of case types. Both factors may influence paramedics’ practice regarding altruism, compassion, and caring behaviours.

The variable nature of ambulance work requires a significant practitioner skillset, one that must be both flexible and ordered, quick and slow, and adaptable to case types and along a continuum. Paramedics must be able to make sense of multiple pieces of information and consider the human needs of the patient while tailoring that person’s care. This is encapsulated in the creation of a holistic practitioner. A well-rounded curriculum needs to cater to these facets to provide graduates who will best serve future patients.

## Data Availability

No new data were created or analysed in this study. Data sharing is not applicable to this article.

## Appendix A

**Table A1:** Summary of Included Articles.

Author(s) and Country of Origin	Year	Design	Study Population and Sample Size	Key Findings	Themes
Ahl and Nystrom Sweden [18]	2012	Explorative, interpretive research—qualitative	20 Interviewees (former patients)	The authors found that pre-hospital caring involves a connectedness between patient and healthcare provider which may have positive or negative patient outcomes. When the public understand the role of the ambulance service in providing advanced care, potential mistrust is eased and aids in a positive care experience.	Themes 1 and 2
Berntsson and Hildingh Sweden [14]	2013	Descriptive, qualitative	17 ambulance mission reports (completed by specialist ambulance nurses during post-graduate studies)	The authors used a theoretical framework to detail four previously identified phases of pre-hospital care (orientation, identification, exploitation, and resolution phase) adding to each phase a set of subcategories—some being repeated during cases; they concluded that phases of the nurse–patient relationship are dynamic and can overlap.	Theme 1
Brainard, C USA [27]	2014	Original commentary	N/A	Used a personal experience to discuss how acts of compassion can have a positive effect on others, particularly on surviving family.	Theme 1
Bremer et al. Sweden [15]	2012	Qualitative	10 emergency medical personnel interviewees	An investigation of EMS experiences and care for grieving family post termination of resuscitation efforts. The authors suggest the development of ethical caring competence can be facilitated through moral education and ethics training, training on appropriate grief responses, and clinical supervision post case.	Themes 1 and 2
Bremer et al. Sweden and Spain [26]	2015	Quantitative	46 ambulance professionals in two countries	A tool (Managerial Values Profile) was adapted for use in two languages and used to measure values held by both Spanish and Swedish ambulance professionals. The authors found that the Spanish favoured social justice while the Swedes favoured moral rights when using values to guide decision making; both groups favoured utilitarianism least—factors that organisations may need to consider this when planning organisation investment.	Theme 1
Brydges et al. Canada [37]	2016	Interpretivist, qualitative	15 semi-structured participant interviews and 79 observations	A study of paramedic roles in community settings from the patient perspective. The author found that paramedics filled dual roles of patient advocacy for health and well-being as well as provision of emergency care.	Theme 2
Carter et al. Canada [38]	2019	Mixed method	49 (part A), patient and family survey/interview and 235/267 (part B pre and post) paramedic surveys	A study of the implementation of a palliative care at home program from the perspective of both patient/family and paramedics. Patient/family satisfaction was high with families noting the professionalism and compassion of paramedics. This approach to palliative care was found to be a positive experience for all parties.	Themes 1, 2 and 3
Dick, T USA [28]	2010	Original commentary	N/A	Explores the practice of kneeling—a practice of necessity that facilitates clinical care or a position of deference, humility. Dick suggests humility acts as a bridge between the person receiving and the person giving care.	Theme 1
Ducar et al. USA [29]	2020	Quantitative	15 emergency service providers	The study profiled the feasibility and impact of a mindfulness intervention program to EMS personnel, finding that the programme could be beneficial. Compassion satisfaction scores increased following the intervention.	Theme 3
Elmqvist et al. Sweden [19]	2010	Qualitative	13 first responder interviewees	Discussion on ‘doing’ and ‘being’ as related to first responder provision of care. Included commentary on education for personal that might arrive before ambulance crew (e.g., police and fire personnel) and education on reflective practice to help individuals recognise how they act in situations of the unknown, ultimately providing the injured person with a safer environment.	Theme 1
Holmberg et al. Sweden [20]	2016	Inductive Qualitative	18 ambulance clinician interviewees	A study focussed on the relationships experienced between ambulance clinicians and patients. Analysis identified one main and three sub-categories, the main being ‘to be personal in a professional role:’ the concepts of the personal and professional are linked in that the clinicians know that they care for case components beyond medical needs. The authors suggest that interpersonal aspects of care are considered in clinician education and development.	Theme 1
Jennings, K UK [32]	2017	Quantitative	32 paramedic students surveyed	The authors identified limited literature regarding emotional labour in paramedicine. Paramedic students surveyed indicated an awareness of their emotions and the perceived need to cover these up. It was suggested that paramedic education would benefit from a stronger understanding of student requirements; this will help incorporate the 6 Cs of nursing into paramedic education.	Theme 3
Kyed, M Denmark [40]	2020	Qualitative	20 ambulance staff interviewees, and a further 9 interviews and observational fieldwork	From the perspective of a male-dominated workforce. Includes commentary on ‘becalming work,’ ‘communication work,’ and ‘bodywork,’ each components of out-of-hospital care.	Theme 1
Lindstrom et al. Sweden [21]	2020	Qualitative	14 patient interviewees	Focussed on the Psykiatrisk Akut Mobilitet (PAM), a Swedish psychiatric emergency response team which focusses on suicide prevention by responding to community-based psychiatric emergencies. The results indicated that involving a patient in their care achieves a person-centred approach and that knowledge of the patient’s vulnerability was key to being able to provide the patient-centred care.	Theme 2
Nordby and Nohr Norway [41]	2012	Qualitative	15 paramedic interviewees	An exploration of paramedic cancer-patient resuscitation and associated ethical dilemmas; the authors explored concepts related to double pressure situations, autonomy in practice, and the role of education in equipping paramedics to resolve ethical dilemmas.	Themes 1 and 3
Rees et al. UK [33]	2018	Qualitative	11 paramedic interviewees	Specific to the paramedic care of those who self-harm. Discusses the complexities of paramedic management of ‘wicked problems,’ a case type that ‘violates’ the ‘sick role’ and the need for training and support for paramedics caring for those who self-harm.	Themes 1 and 3
Rees et al. UK [34]	2017	Qualitative	11 paramedic interviewees	An exploration of the paramedics’ perceptions and experiences in providing care for those who self-harm. Suggests an ‘urgent need’ to add findings from the study to guidelines and education on caring for those who self-harm	Theme 1
Rees et al. UK [35]	2015	Qualitative	Metasynthesis of qualitative research (12 articles)	An intended exploration of the literature related to the perceptions of paramedic and emergency care for those who self-harm. Such literature is limited, and scope exists for further research in this field. Ultimately self-harm was described as a common but professionally, clinically, and socially complex interaction.	Themes 1, 2 and 3
Rees et al. UK [36]	2014	Quantitative	Systematic review of quantitative literature (16 articles)	An investigation of the understanding of paramedic and emergency workers’ perceptions of caring for those who self-harm. One of the 16 articles was specific to paramedic care; the authors found limited evidence of training applicable to self-harm—training was shown to improve knowledge, confidence, and attitude (more positive) in those caring for patients who self-harm.	Themes 1 and 2
Rubin, M USA [31]	2010	Original commentary	N/A	A short exploration on how paramedics might approach cases with compassion based on the author’s observation of a colleague’s caring work. Suggests a few simple actions (such as the patient’s choice of music) that could brighten a patient’s experience.	Theme 1
Sandstrom et al. Sweden [22]	2017	Qualitative	13 patient interviewees	An exploration of the patients’ experiences of helicopter emergency medical services (HEMS), the provision of personal care, and how patients can trust and surrender themselves to caregivers.	Themes 1 and 2
Sundstrom and Dalberg Sweden [23]	2012	Qualitative	11 ambulance carer observations, notes, and interviews	Discussed how care outcomes affect patient security; conversations with patients comfort the patient while providing reliable information. Pre-case information can provide grounds for preparedness; nonetheless, paramedics need to be adaptable should situations differ.	Theme 1
Touchstone, M USA [31]	2010	Original commentary	N/A	Explored the concept of the paramedics’ core values and how these can form a foundation for action. Discussed honesty, integrity, caring, and compassion.	Theme 3
Williams et al. Australia [39]	2013	Quantitative	94 undergraduate paramedic student surveyed	Study explored student paramedic empathy levels. Explored the advantages of empathic care and how empathy might be integrated into the paramedic curriculum.	Themes 1, 2 and 3
Wireklint Sundstrom et al. et al. Sweden [24]	2019	Systematic Review	78 studies	The authors identified 78 studies with a focus on caring science; most of these studies originated in Sweden. The authors conclude that there is limited exploration of caring science from an ambulance-patient perspective.	Themes 1, 2 and 3
Wireklint Sundstrom and Dalbert Sweden [25]	2011	Qualitative	11 ambulance carer observations, notes, and interviews	The authors concluded that inclusion of the patient perspective and an openness regarding their lifeworld should form the backbone of care interactions. Whereas a focus on general patient behaviours and medical conditions may create a care barrier.	Theme 1
Young, R Canada [5]	2014	Original commentary	N/A	Outlined the components and benefits of compassion and suggested that compassion is “a trait of the altruistic.” Discussed the practice of and education about compassion. Also presented the downside of compassion such as compassion fatigue.	Themes 1 and 2
